# Mechanical removal of epithelial hyperplasia leads to successful treatment of irregular astigmatism

**DOI:** 10.1186/s12886-023-02870-z

**Published:** 2023-03-27

**Authors:** Mª Victoria de Rojas Silva, Juan Álvarez de Toledo, Adrián Tobío Ruibal

**Affiliations:** 1 Victoria de Rojas Instituto Oftalmológico - Policlínica Assistens, A Coruña, Spain; 2 Clínica Oftalvist, Barcelona, Spain

**Keywords:** Irregular astigmatism, Epithelial hyperplasia, Epithelial mapping, Pseudokeratoconus

## Abstract

**Background:**

Corneal epithelium remodeling in response to changes in the anterior corneal surface (keratoconus, corneal refractive surgery) is well-documented in the literature. However, several conditions may induce a different behavior of the epithelium, in which focal areas of epithelial thickening induce irregular astigmatism. This case report presents a highly unusual case of irregular astigmatism induced by an epithelial hyperplasia of unknown etiology, which was treated by the mechanical removal of only the epithelium.

**Case presentation:**

A 29-year-old woman underwent implantable collamer lens implantation to correct myopia. The patient provided written informed consent. The procedure was uneventful in both eyes. Twenty months later, she complained of decreased visual acuity in the left eye (uncorrected distance visual acuity (UCDVA) was 20/30; corrected distance visual acuity was 20/20 with + 1.00 -2.25 × 170). Corneal topography revealed a nasal steepening in the left eye. Although the corneal thickness map was normal, epithelial thickness mapping revealed a localized nasal area of epithelial hyperplasia in the left eye that matched the area of steepest curvature. Slit lamp examination showed a total clear cornea with no signs of abnormality. The patient´s medical history was unremarkable and a case of epithelial hyperplasia of unknown etiology, without active inflammation, was considered. The decision was made to perform a mechanical removal of the corneal epithelium after application of diluted alcohol. One month after the procedure, the topography of the epithelized cornea showed a regular bow tie pattern and UCDVA improved to 20/20. No recurrence of the epithelial hyperplasia was detected after twenty-one months.

**Conclusions:**

Focal epithelial hyperplasia may induce irregular astigmatism. Epithelial thickness mapping is a very helpful technological tool to assess cases with irregular topography. De-epithelization as an isolated procedure may be useful for the successful management of these cases. Further research is required to understand the mechanism that triggers the spontaneous development of a focal epithelial hyperplasia.

## Background

Previous studies have described cases of irregular astigmatism caused by changes in epithelial thickness leading to corneal topographic changes. Although most of these cases occurred after superficial laser refractive ablation [1], other instances showed keratoconus-like topographic changes due to inferior inhomogeneous epithelial thickening [2–4].

Corneal epithelium remodeling in response to changes in anterior corneal curvature or an underlying stromal pathology is well-documented in the literature. This remodeling has been studied in cases of ectatic conditions such as keratoconus, in which the epithelium thins over the point of maximum corneal curvature and thickens at the base of the cone, thereby masking the real extent of anterior steepening [5]. On the other hand, the center of the corneal epithelium thickens to compensate for flattening induced by myopic laser vision correction. This induces myopic regression after laser-assisted in situ keratomileusis correction, where the epithelium thins over the hills and thickens over the valleys of an irregular stroma in order to smooth and mask stromal irregularities [3].

However, in some cases, epithelium behaves differently, with focal areas of epithelial thickening inducing irregular astigmatism. In these cases, keratoconus-like topography patterns can be observed, wich are associated with localized epithelial thickening in the steepest area, leading to irregular astigmatism. Different conditions can cause this epithelial hyperplasia in unoperated corneas and this behavior has been observed after re-epithelization following laser photorefractive keratectomy [1–4, 6–9].

The ability to measure corneal epithelial thickness and the characterization of its behavior in response to changes in the corneal architecture have become objects of increased interest in clinical practice. Epithelial mapping plays a crucial role in clinical practice, as it can not only differentiate normal and suspicious patterns, but also inform refractive surgery decisions, and guide the treatment of corneal surface disorders [1–10].

This case report presents a highly unusual case of irregular astigmatism induced by an epithelial hyperplasia of unknown etiology, which was successfully treated by mechanical removal of the epithelium only.

## Case presentation

**Fig. 1:**
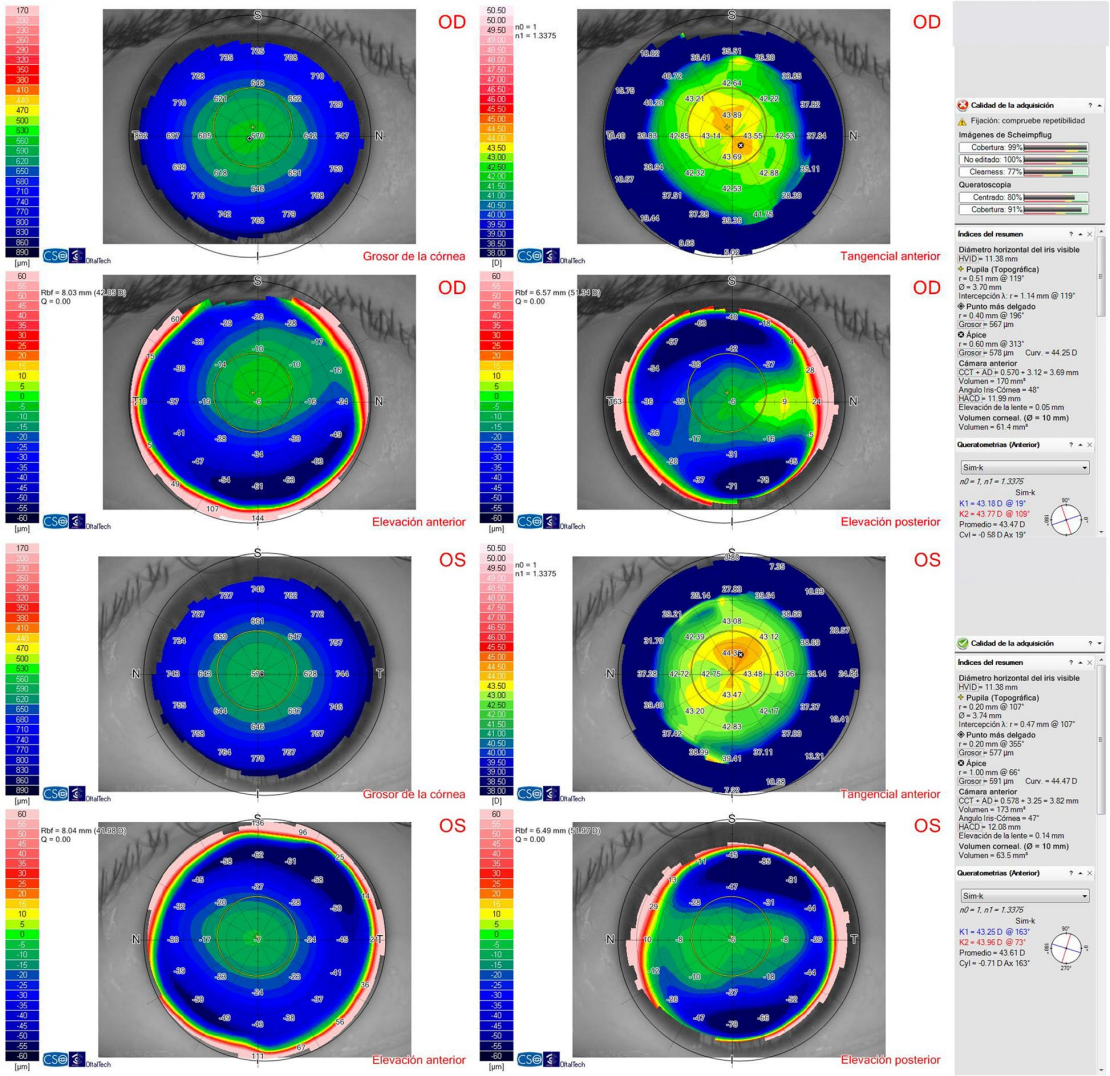
Preoperative corneal topography showing a regular topography within normal limits in both eyes

**Fig. 2:**
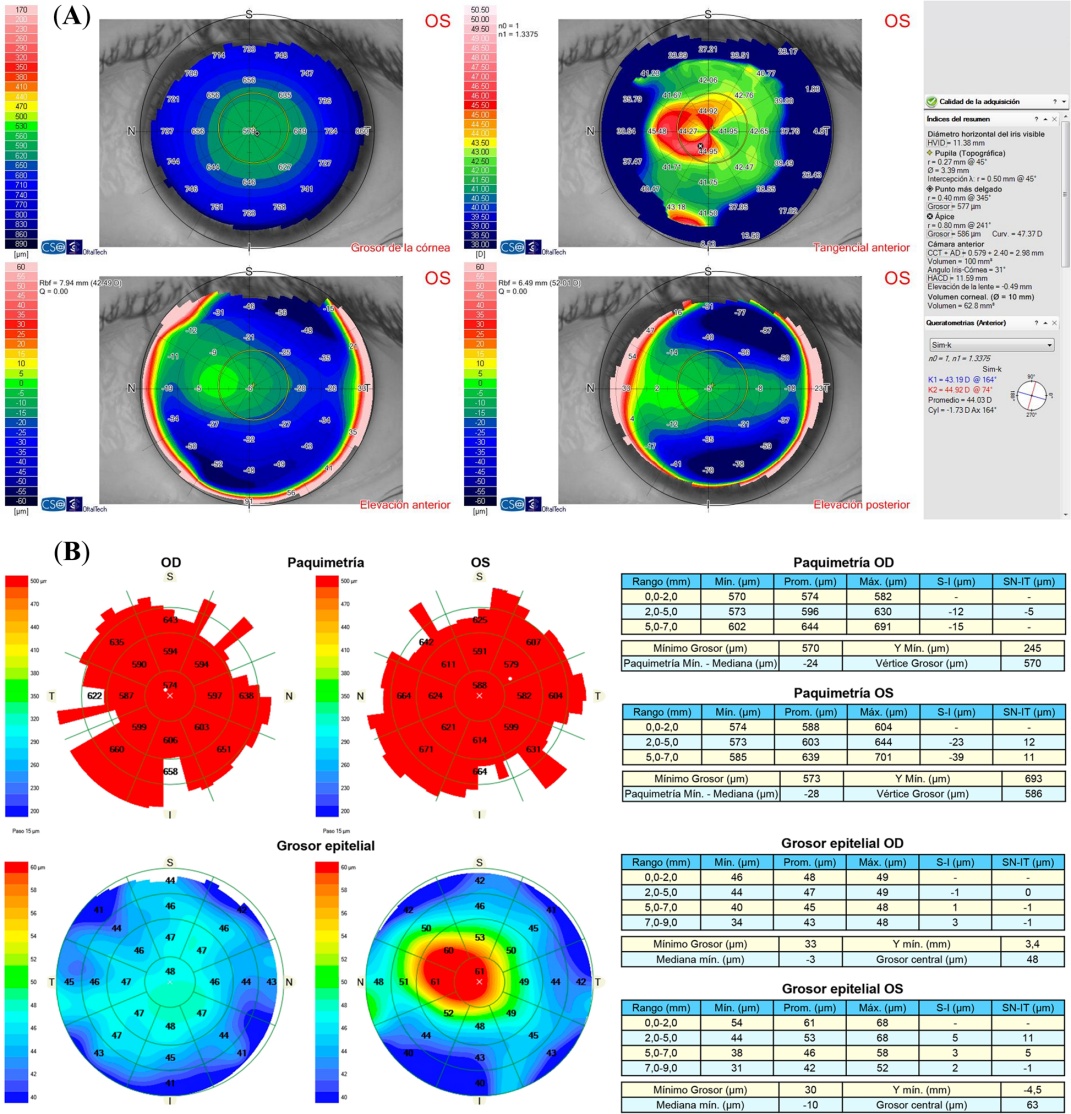
**A** Corneal topography of the left eye twenty months after ICL implantation showing irregular corneal astigmatism with a nasal D steepening pattern. **B** Epithelial map of both eyes revealing a normal pattern in the right eye and a nasal focal area of epithelial hyperplasia that was coincident with the area of steepest curvature of the topography in the left eye

A 29-year-old woman underwent implantable collamer lens (ICL) (STAAR Surgical, Monrovia, CA, USA) implantation to correct myopia in both eyes (right eye -7.00 -0.25 × 55 = 20/20 left eye -7.00 -0.50 × 155 = 20/20). Preoperative topography obtained with Sirius (Oftaltech, Construzione Strumenti Oftalmici, Florence, Italy) showed a symmetric bow tie pattern in both eyes (Fig. 1). This device, which combines a 360-degree rotating Scheimpflug camera with a 22 ring Placido disc, provides data regarding the tangential and axial curvature of the anterior and posterior corneal surfaces, the global refractive power of the cornea, corneal pachymetry mapping and wavefront analysis. Endothelial cell density before surgery was 2.660 and 2.667 cells/mm^2^ in the right and left eye respectively. Three months after surgery, uncorrected distance visual acuity (UCDVA) was 20/20 and 20/25 in the right and left eye respectively. Corrected distance visual acuity (CDVA) in the left eye improved to 20/20 with -0.75 × 160º. Vault was 650 μm and 464 μm in the right and left eye respectively. Twenty months after surgery, she complained of decreased visual acuity in the left eye. UCDVA was 20/20 in the right eye and 20/32 in the left eye, which improved to 20/20 with + 1.00 -2.25 × 170º in the left eye. Corneal topography was normal in the right eye, but nasal steepening was observed in the left eye (Fig. 2A). Tomographic keratoconus indices were within normal limits, as were posterior elevation, the corneal thickness spatial profile and percentage thickness increase curves. The corneal thickness map was normal, with a thinnest point of 577 μm. Epithelial mapping was normal in the right eye but a nasal focal area of epithelial hyperplasia (68 μm) was observed in the left eye, coinciding with the area of steepest curvature (Fig. 2B). This area was 17 μm, 24 μm, 25 μm and 22 μm thicker than the surrounding epithelium, which measured 51 μm, 44 μm, 43 μm and 46 μm in the nasal, temporal, inferior and superior zones of the 5–7 mm diameter ring surrounding the hot spot.

**Fig. 3:**
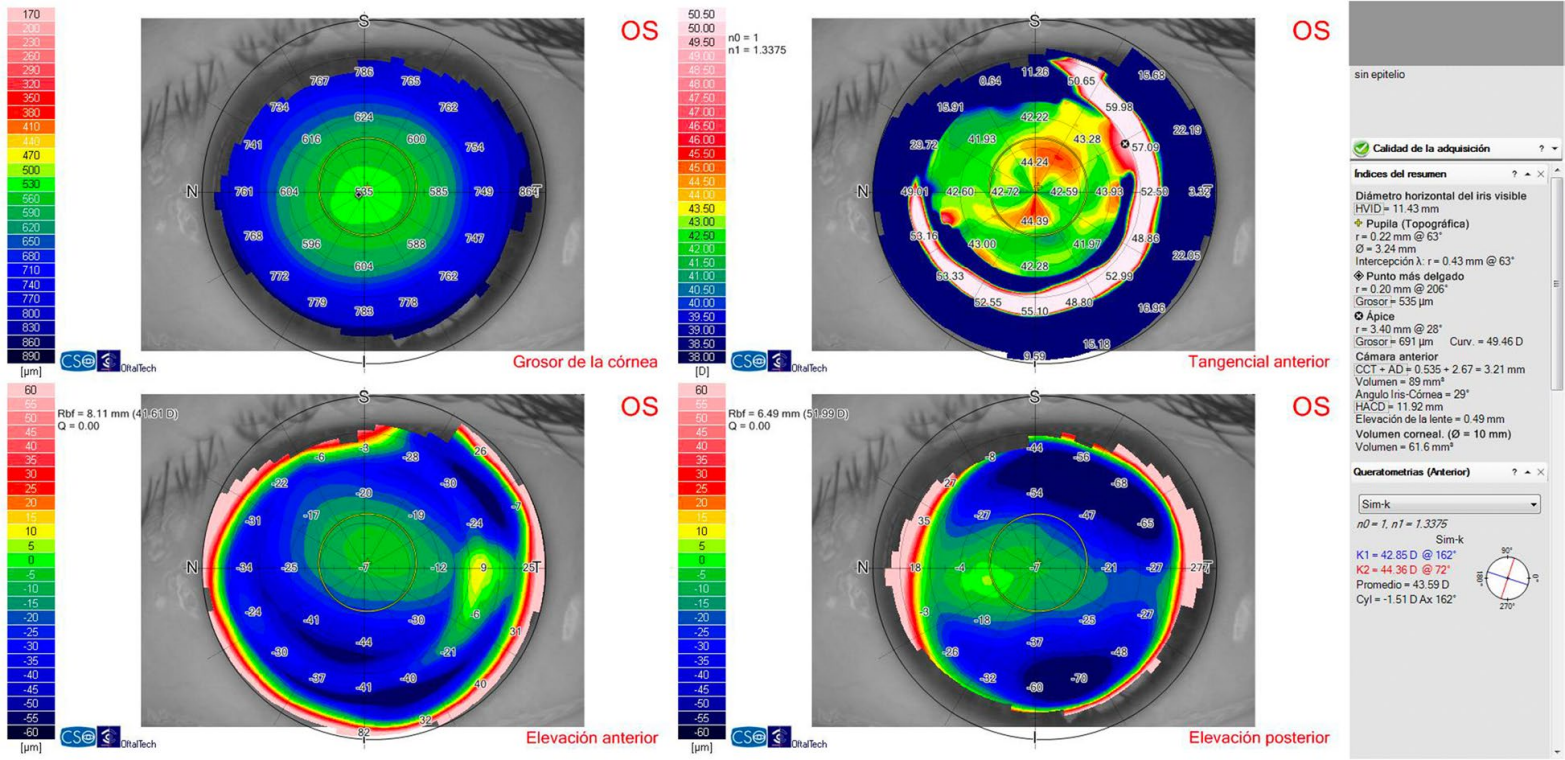
Intraoperative topography of the Bowman layer immediately after the removal of the corneal epithelium showing a perfectly regular symmetric bow tie pattern in the left eye

**Fig. 4:**
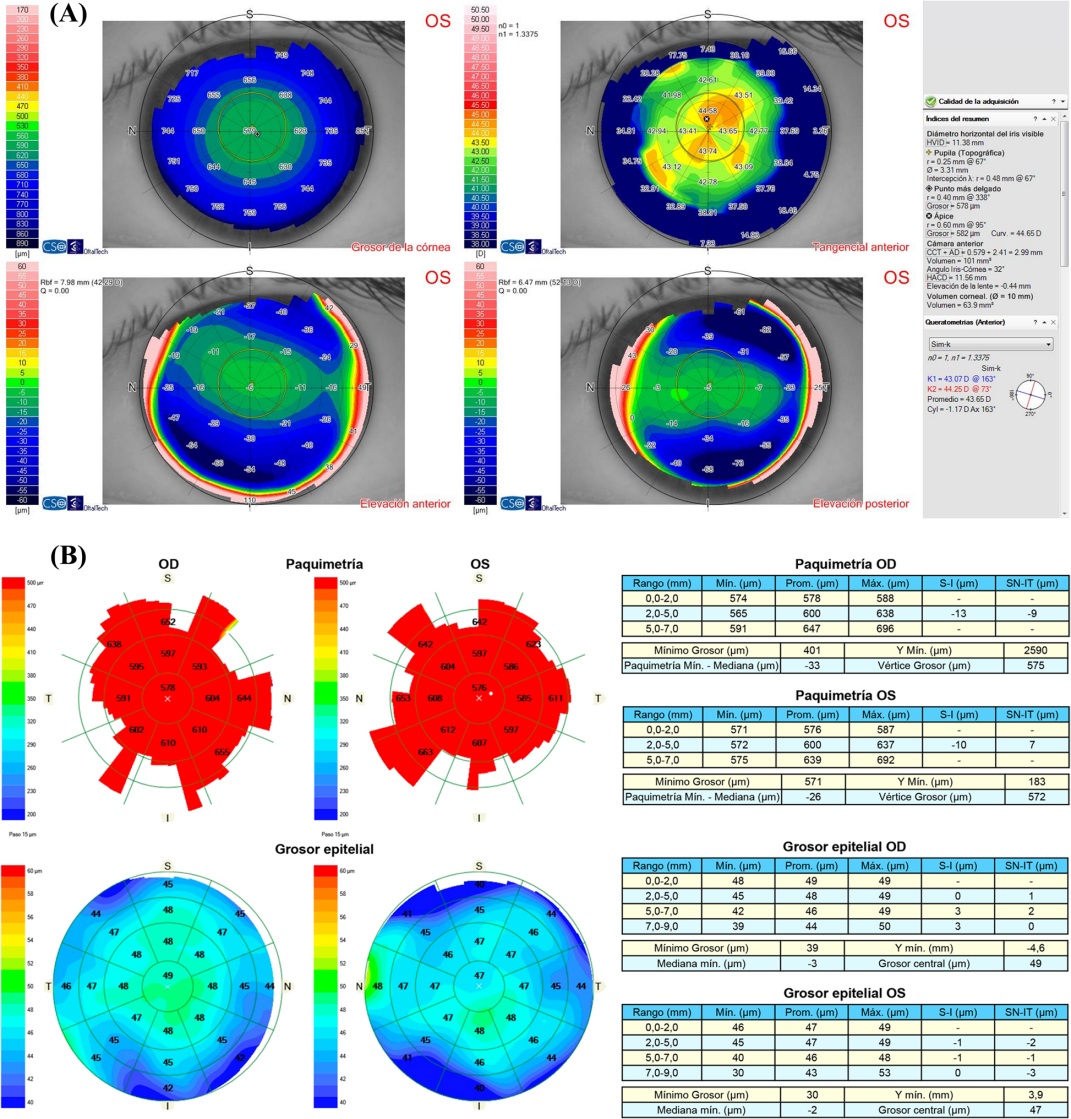
**A** Topography of the epithelized cornea showed a regular bow-tie pattern one month after epithelial removal in the left eye. **B** Epithelial mapping revealed a normal pattern eight months after epithelial removal in the left eye

**Fig. 5:**
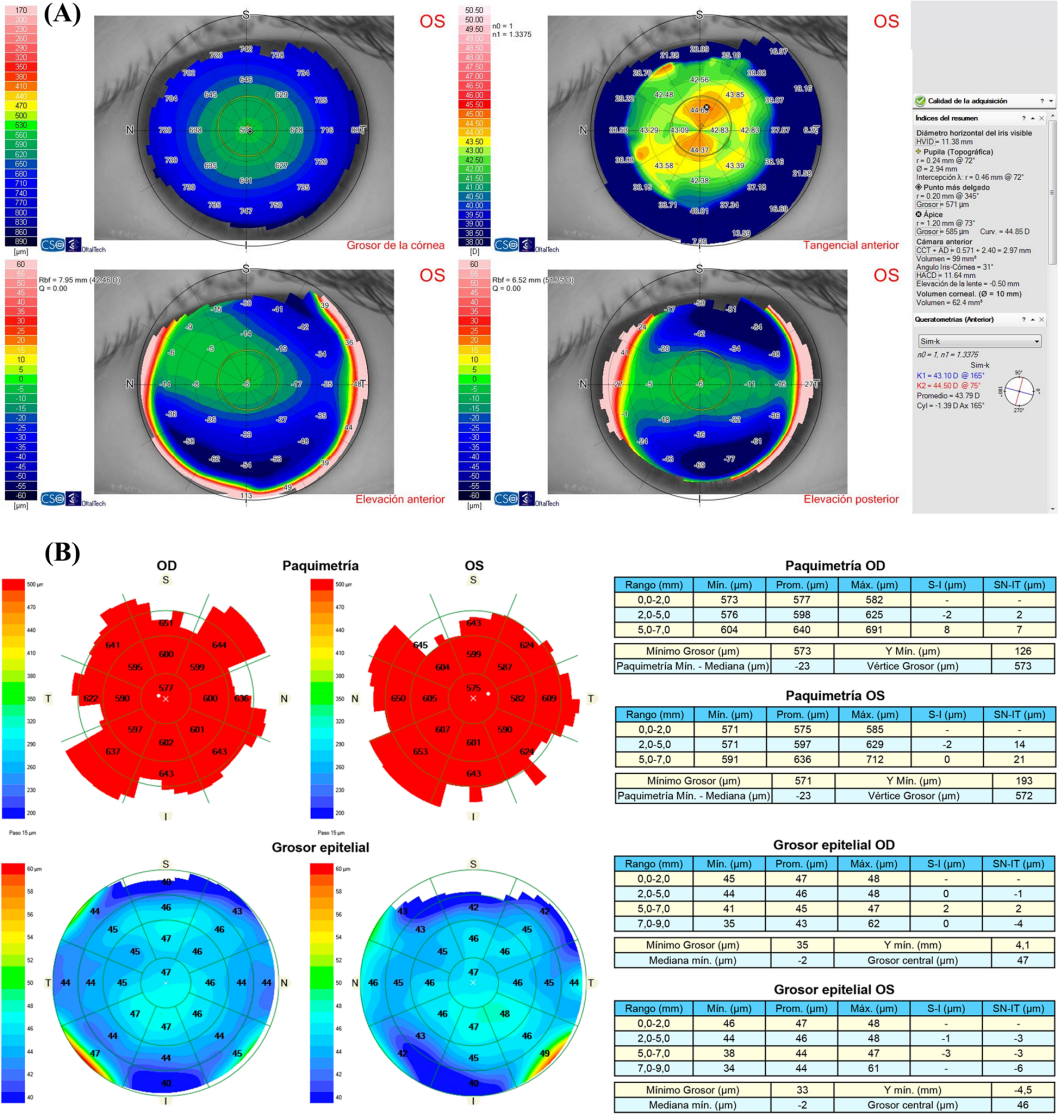
**A** Corneal topography showed a regular bow-tie pattern 21 months after epithelial removal in the left eye. **B** Epithelial mapping revealed a normal pattern 21 months after epithelial removal in the left eye

Slit lamp examination showed a totally clear cornea with no signs of abnormality: no signs of epithelial basement membrane dystrophy (either by negative staining or retroilumination), blepharitis, keratitis, corneal edema, haze, meibomian gland dysfunction, Salzmann degeneration or intraepithelial neoplasia were present. The appearance of the iris was normal and no anterior chamber cells or flare were detected. Intraocular pressure was 14 mmHg in both eyes and the retina was normal. Anterior chamber depth was 3.41 mm in the right eye and 3.39 mm in the left eye. Vault was 582 μm and 410 μm in the right and left eye respectively. Endothelial cell density was 2.509 and 2.586 cells/mm^2^ in the right and left eye respectively. No signs of epithelial basement membrane dystrophy were detected upon anterior segment optical coherence tomography examination (AS-OCT) (Cirrus 500 HD anterior segment module, Carl Zeiss Meditech AG, Jena, Germany). The patient reported no history of ocular trauma, systemic disease, or medication. She had not been wearing contact lenses. She referred to rubbing her eyes, though very rarely. The patient was instructed to completely avoid eye rubbing and artificial tears were prescribed, but irregular astigmatism induced by epithelial hyperplasia not only persisted, but slowly increased over the following six months (from -1.53 × 164 twenty months after ICL implantation to -2.01 × 167 six months later). A case of epithelial hyperplasia without active inflammation of unknown etiology was considered and the decision was made to perform the mechanical removal of the corneal epithelium after the application of diluted alcohol. The epithelium was carefully peeled off with a blunt spatula after the application of a 20% alcohol solution for 20 s on the 8.0 mm central corneal zone. The speculum was then removed and the patient was asked to sit at the topography instrument. The topography of the Bowman layer was analyzed immediately after the removal of the corneal epithelium and showed a perfectly regular symmetric bow tie pattern (Fig. 3), which confirmed that the focal epithelial hyperplasia was the cause of the irregular pattern of corneal topography. One month later, topography of the epithelized cornea showed a regular bow tie pattern (Fig. 4A) and UCDVA improved to 20/20 and CDVA was 20/20 with -0.50 × 160º. Eight months after surgery, UCVA was 20/20 and CDVA 20/20 with -0.5 × 160º and epithelial mapping revealed a normal pattern in both eyes (Fig. 4B). In the last follow-up visit, twenty-one months after epithelial removal, UCVA was 20/20 and CDVA was 20/20 with -0.5 × 165º, and no recurrence of irregular astigmatism or hyperplasia was observed (Fig. 5).

## Discussion and conclusions

The case described in this paper demonstrates how corneal irregular astigmatism can be caused by epithelial hyperplasia and how it can be managed by epithelium removal only. It also highlights the importance of epithelial mapping for diagnosis and management decision-making in this and similar cases.

Epithelial mapping revealed a nasal focal area of epithelial hyperplasia (68 μm) in the left eye that coincided with the area of steepest curvature. Reinstein et al. reported that in normal corneas, epithelium mean central thickness was 53,4 μm, with the superior epithelium being 5,7 μm thinner than the inferior epithelium and 1,2 μm thicker in the nasal than in the temporal regions [5]. Thus, the epithelial pattern in our case obviously did not correspond to that of a normal cornea.

The nasal location of the corneal steepening, together with the normal pachymetry map, normal posterior elevation and tomographic indices, as well as the corneal thickness spatial profile and the percentage of thickness increase curves, and the negative result of the keratoconus algorithm ruled out keratoconus. The fact that epithelial hyperplasia coincided with nasal steepening contradicted a diagnosis of keratoconus, in which epithelial thinning over the cone would be expected. Contact lens-induced corneal warping usually shows epithelial hyperplasia over the steep area of corneal curvature [9]. However, our patient had not used contact lenses for five days before surgery, and preoperative topography was normal. In fact, she had not worn contact lenses since the ICL implantation, meaning she had gone 20 months without contact lenses when she noticed the decrease in visual acuity in her left eye.

Focal epithelial hyperplasia with accompanying corneal steepening mimicking keratoconus can be caused by different conditions: inflammatory conditions (blepharitis, Meibomian gland dysfunction, viral keratitis), neoplasic conditions (preclinical corneal intraepithelial neoplasia), degenerative conditions (Salzmann nodular degeneration, peripheral hypertrophic subepithelial corneal degeneration, epithelial basement membrane dystrophy), or traumatic conditions [2, 4, 6–9]. All these conditions were ruled out after a careful slit lamp examination coupled with AS-OCT exams. Recently, Levy et al [10] showed that epithelial map pattern recognition combined with a quantitative analysis of epithelial thickness, is relevant for the diagnosis of ocular surface diseases and for distinguishing various diseases from each other. They described 14 epithelial map patterns, some of which were related to the diagnosis of certain ocular surface pathologies. However, none of these patterns fit with the pattern observed in our patient for whom endothelial cell density and ICL vault were within normal limits in both eyes and no endothelial alteration or corneal edema was detected. In addition, the evolution of the case was observed over the course of six months, which would have allowed any possible pathology to manifest itself.

The patient developed this irregular astigmatism after ICL implantation. One may speculate as to whether the irregular corneal astigmatism may have been induced by the corneal incision. The nasal area location of corneal steepening does not support the hypothesis of a surgically-induced alteration, nor does the presentation 20 months after ICL implantation. Had the surgical incision played a role in the nasal steepening, it would have become manifest soon after surgery. An alteration of epithelial mapping has been reported after cataract surgery with a slight increase in central epithelial thickness [11]. In this case, there was a paracentral hot spot on the epithelial map, not a central one. No report dealing with epithelial mapping alteration after ICL has been published.

We also speculated as to whether the focal hyperplasia was due to eye rubbing. The patient reported rubbing her eyes though very rarely. There are few publications regarding the effects of eye rubbing on the corneal epithelium [12–14]. No significant differences in epithelial thickness were found after eye rubbing in one paper [12], while epithelial thinning changes were described in two other studies [13, 14]. None of these epithelial thinning map patterns are consistent with the localized epithelial thickening we observed in our patient and, in addition, the hot spot of epithelial hyperplasia persisted without changes after six months without eye rubbing.

Due to the epithelial origin of the irregular astigmatism, we decided to proceed with solely epithelial removal first. We performed intraoperative topography with the aim of confirming the epithelial origin of the irregular astigmatism. Topography immediately after removing the epithelium showed a perfect bow tie pattern, further supporting the epithelial origin of the hot spot observed in topography.

A recently-published paper illustrated a similar scenario, though in that case, the patient had a history of contact lens intolerance and the cause of the irregularity was related to corneal warpage according to some, but not all of the panelists [6]. There were no other corneal alterations that could have been the cause of the irregularity and corneal ectasia had been excluded since the pachymetry map was normal, different keratoconus algorithms ruled out keratoconus and, in addition, as in our case, and unlike in keratoconus, the area of epithelial hyperplasia coincided with the area of corneal steepening. Unlike our case, this paper did not provide intraoperative topography but the management of the case was the same, with epithelial removal only, which successfully reverted the condition. It is our opinion, and that of other authors, that epithelial removal only can be used to treat epithelial hyperplasia in the absence of underlying stromal irregularity [1, 6]. Although some of the panelists in the previous paper [6] suggested and agreed with this approach, and Hwang [1] proposed the same procedure for the treatment of epithelial hyperplasia in the absence of any underlying stromal irregularity, we are not aware of any published series of cases evaluating the results of this procedure to treat similar cases. Our case does provide further supports for this approach.

The main limitation of this report is that the information provided here was obtained from an isolated case. In addition, a longer follow-up would be required to evaluate whether the epithelial alteration could relapse.

In conclusion, focal epithelial hyperplasia may induce irregular astigmatism. Epithelial mapping is a very useful technology to assess cases with irregular topography. De-epithelization as an isolated procedure may be useful for the successful management of these cases. Further research is required to fully understand the mechanism that triggers the development of a focal area of epithelial hyperplasia in an otherwise normal eye.

## Data Availability

The data that support the findings of this study were obtained from the medical records of the patient and are not publicly available. The data are available on reasonable request from the corresponding author, [MVR].

## Abbreviations

ICL: Implantable Collamer Lens
UCDVA: Uncorrected Distance Visual Acuity
CDVA: Corrected Distance Visual Acuity
AS-OCT: Anterior Segment Optical Coherence Tomography
